# Metformin: a metabolic modulator

**DOI:** 10.18632/oncotarget.14794

**Published:** 2017-01-22

**Authors:** Federico Pietrocola, Guido Kroemer

**Affiliations:** ^1^ Gustave Roussy Comprehensive Cancer Institute, Villejuif, France; ^2^ INSERM, U1138, Paris, France; ^3^ Equipe 11 labellisée par la Ligue Nationale contre le Cancer, Centre de Recherche des Cordeliers, Paris, France; ^4^ Université Paris Descartes/Paris V, Sorbonne Paris Cité, Paris, France; ^5^ Université Pierre et Marie Curie/Paris VI, Paris, France; ^6^ Metabolomics and Cell Biology Platforms, Gustave Roussy Cancer Campus, Villejuif, France; ^7^ Pôle de Biologie, Hôpital Européen Georges Pompidou, AP-HP, Paris, France; ^8^ Department of Women’s and Children’s Health, Karolinska University Hospital, Stockholm, Sweden

**Keywords:** aging, autophagy, biguanides, caloric restriction, cancer

## Abstract

Recent findings have shed new light on the mechanisms of action through which biguanides exert their anti-aging and cytostatic effects in *Caenorhabditis elegans* and human cell lines. The drop in energy charge resulting from the metformin mediated inhibition of mitochondrial activity affects the function of the nuclear pore complex, blocks mTOR signaling and enhances the expression of ACAD10. Whether the inhibition of this pathway is truly responsible for the anti-diabetic and cancer effects of the drug in mammals remains to be established.

The old drug class of biguanides represents the gold standard treatment for type-2 diabetes (T2D). The glucose lowering and insulin sensitizing effects of these molecules account for their therapeutic use in the context of metabolic syndromes. However, the exact mode of action of these drugs remains largely elusive at the molecular level.[1] Of note, the growing interest of investigators towards a full comprehension of the molecular mode of action of metformin goes far beyond its anti-diabetic functions. In the recent past, epidemiological and fundamental research studies have indeed disclosed the therapeutic potential of biguanides in the prevention and treatment of various cancer types. [2] The hyperactivation of nutrient signaling pathways (in particular the insulin/mTORC1 axis) resulting from overfeeding or genetic aberrations sets the metabolic grounds for accelerated aging and malignant transformation.[3] Metformin administration partially corrects these defects and additionally enhances health- and lifespan in different model organisms, while it reduces the incidence of cancers in rodent models as well as in patients.[3]

In their recent work, Wu and colleagues report that metformin elicits a dose-dependent cytostatic effect in *C. elegans*.[4] Through a genetic screening using a siRNA library targeting metabolic/bioenergetic genes, the authors identified proteins involved in the sensitization or resistance of worms to biguanides. As previously described, metformin retains a primarily mitochondrial tropism due to its inhibitory effects towards complex I. Indeed, genes involved in metformin sensitization encompassed a mitochondrial complex 1 subunit and the main subunit of the pyruvate dehydrogenase complex that is responsible for the generation of mitochondrial acetyl coenzyme A (CoA). This latter observation is in line with the recently published observation that metformin effectively reduces acetyl CoA biosynthesis and consequently the acetylation of histone proteins.[5] Conversely, the knockdown of the mitochondrial beta-oxidation regulator ACAD10 conferred the highest degree of resistance to high-dose metformin treated nematodes. This enzyme, whose variants have been associated with human T2D and insulin resistance [6] potentially represents a hub of key cellular processes, as supported by the fact that it co-immunoprecipitated with proteins involved in ribosomal, mitochondrial and cell growth functions; *de facto*, knockdown of ACAD10 abolished the lifespan-extending effect of metformin in *C. elegans*.[4]

As an additional proof of a direct link between the inhibition of mitochondrial function and ACAD10 expression, Soukas’ group demonstrated that silencing of the mitochondrial respiration component *gas-1* or pharmacological inhibition of mitochondrial complex 1 by rotenone caused an increase in the cytoplasmic levels of ACAD10 protein. Moreover, a comparable effect was achieved upon treatment of nematodes with the mTORC1 inhibitor rapamycin but not with the AMPK activator AICAR. [4] This result, along with the observation that metformin retained the ability to elevate ACAD10 levels upon AMPK silencing, corroborated the notion that AMPK activation, resulting from reduced mitochondrial ATP production, is dispensable for the lifespan extension effect of biguanides [7] (Figure 1).

**Figure 1:**
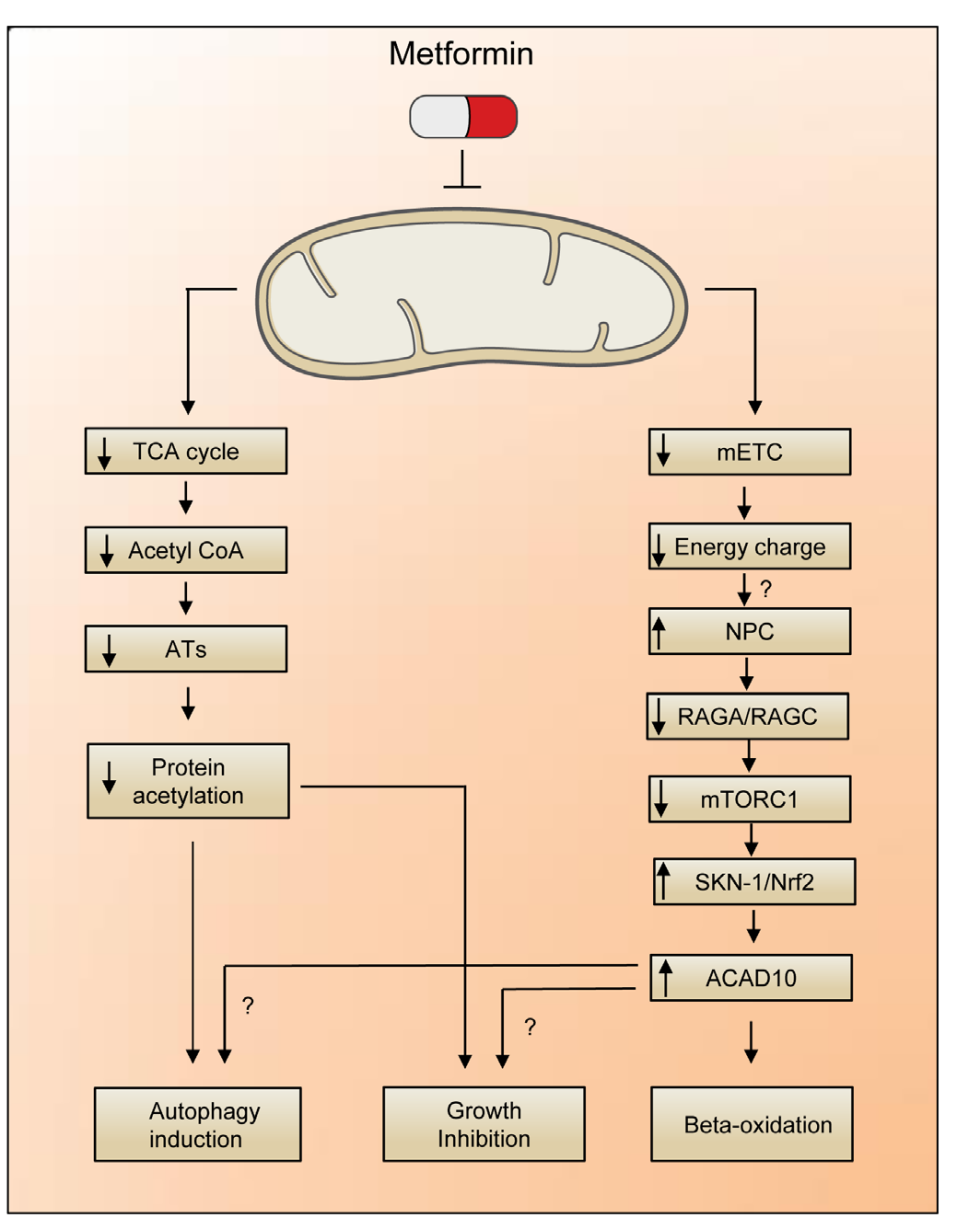
AMPK-independent mechanisms underlying metformin-induced lifespan extension A reduction in energy charge, as consequence of the metformin-mediated inhibition of mitochondrial electron transfer (mETC) leads to the limited shuttling of RAGA/RAGC complex trough the nuclear pore complex (NPC). The block of mTORC1 signaling unleashes the activity of SKN1/Nrf2, a transcription factor that stimulates the expression of ACAD10. Alternatively, the reduction in Acetyl CoA levels (correlating with a reduced activity of acetyltransferases, ATs) may promote a decrease in protein acetylation with consequent upregulation of autophagy and epigenetic reprogramming of the cell.

Noteworthy, a forward genetic screening revealed that missense mutations in the nuclear pore complex (NPC) components *npp-3* and *npp-21* (leading to impaired NPC formation and to abnormal nucleo-cytosolic shuttling) rendered worms resistant to metformin and rotenone-induced growth inhibition and ACAD10 downregulation. Conversely, rapamycin still retained the capacity to elevate ACAD10 levels in these settings. The ability of metformin and rotenone to limit mTORC1-mediated phosphorylation of p70 ribosomal S6 kinase also relied on an intact NPC. Altogether, these findings delineate a novel hierarchic pathway in which inhibition of the mitochondrial function exerted by biguanides is epistatic to NPC, whereas mTORC1 acts downstream of NPC to control ACAD10 expression.[4] Demonstrating the probable evolutionary conservation of this route, the authors showed that NPC inhibition and ACAD10 upregulation were both required for limiting growth of human cancer cell lines treated with metformin. Although this aspect deserves further characterization, Wu et. al. showed that the reduction in energy charge, rather than mitohormetic phenomena, represented the causal nexus between mitochondrial complex I inhibition and NPC function.[4]

Previous reports have shown that phenformin retains the ability to reduce passive transport through NPC and to inhibit mTORC1 in a manner that depends on Rag GTPase (RAG) heterodimers.[1],[7] Wu and colleagues hypothesized that phenformin limits mTORC1 activation by restraining the nucleo-cytosolic transit of RAGs through the NPC. To achieve the full capacity to activate mTORC1 at the lysosomal surface, the RAG subunit RAGA (in its GTP binding conformation) needs to form a heterodimer with a GDP-binding RAGC.[8] The authors revealed that the formation of a transient inactive RAGA-GDP/RAGC-GTP heterodimer is required for the passive translocation of RAGC through the NPC. Once in the nucleus, RAGC acquired its GDP-binding state through interaction with folliculin, a previously identified protein with GTPase activating protein (GAP) activity towards RAGC. Importantly, pretreatment with phenformin (or expression of a 3xGFP-RAGC fusion protein that cannot translocate through the NPC due to its elevated molecular size) prevented nucleo-cytosolic shuttling and led to mTORC1 inhibition and ACAD10 expression. [4]

According to a prior report, the transcription factor Skn-1/Nrf-2, which regulates the transcription of antioxidants/cytoprotective genes and is repressed by mTORC1, is indispensable for the metformin-mediated increase in lifespan observed in *C. elegans*.[9] Soukas’ group now provides evidence that ACAD10 expression triggered by metformin was partially reduced upon Skn-1 silencing, explaining a possible role of this factor in the pro-health effects of metformin. [4]

The unexpected link between the bioenergetic crisis elicited by metformin-mediated inhibition of mitochondrial activity and reduced nucleo-cytosolic shuttling of cellular proteins opens exciting perspectives on the mode of action of biguanides. It appears indeed plausible that metformin leads to an overall, rather than selective, reduction in the transit of proteins through NPCs. The identification of proteins whose subcellular localization is affected by metformin may lead to the characterization of new therapeutic targets acting in the pathway elicited by biguanides. Similarly, screening of molecules able to mimic the ability of biguanides to restrain passive shuttling through the NPC may prompt the identification of new drugs with antidiabetic and antineoplastic properties.

The direct consequence of mTORC1 inhibition is the activation of cytoprotective autophagy, an event that is sufficient to extend longevity in *C. elegans* and other model organisms. [10],[11] Caloric restriction or treatment with agents mimicking molecular features of dietary restriction, so called ‘caloric restriction mimetics’ (CRMs) prolongs lifespan of various model organisms depending on autophagy activation.[11] The finding that metformin reduced mitochondrial acetyl CoA production suggest that this molecule acts as a CRM, knowing hat the unifying principle of CRMs is that they reduce protein acetylation.[11] However, it remains to be established whether the mode of action of metformin (and that of its direct target ACAD10) depends on the induction of bulk autophagy or rather specific autophagic pathways such as mitophagy.

Whether the mTORC1/ACAD10 axis represents the primary target of metformin *in vivo* remains to be explored. In this context, the identification of confounding non-cell autonomous effects is of cardinal importance to achieve a full comprehension of the mode of action of biguanides. As an example, the anti-aging role of metformin has previously been correlated with modifications of the composition of the gut microbiota.[12] Moreover, the recent literature suggests that the anticancer properties of metformin rely on the activation of immune system and the extinction of protumorigenic inflammation.[13] Hence, it will be important to establish the molecular links between the cell-autonomous and organism-wide effects of biguanides.
